# Hereditary angioedema prevalence and satisfaction with prophylaxis in South Australia

**DOI:** 10.1016/j.waojou.2024.100918

**Published:** 2024-06-18

**Authors:** Alexander Troelnikov, Karen Milburn, Pravin Hissaria, Thanh Thao (Adriana) Le, William Smith

**Affiliations:** a Royal Adelaide Hospital, South Australia, Australia; b SA Pathology, South Australia, Australia; c Flinders University, South Australia, Australia; d University of Adelaide, South Australia, Australia; e Royal Hobart Hospital, Tasmania, Australia

**Keywords:** Angioedemas, Hereditary, Epidemiology, Health-related quality of life, Angioedemas, Hereditary/therapy

## Abstract

**Background:**

Hereditary angioedema (HAE) due to deficiency of C1 Inhibitor (C1INH-HAE) is a rare, unpredictable and potentially fatal genetic disorder. There are relatively few systematic population prevalence studies, with reports from various countries of between 1 in 20,000 and 1 in 150,000. and no Australian data. The therapeutic landscape for HAE has changed dramatically in recent years with a focus on highly effective prophylaxis, with the aim of total suppression of angioedema and achievement of a normal life.

**Objectives:**

Epidemiological survey of HAE in South Australia, with description of patient characteristics, quality of life and treatment, with a focus on prophylaxis.

**Methods:**

Case ascertainment was conducted over 18 months from January 2021 to July 2022, using a range of approaches with the aim of identifying all people with C1INH-HAE in South Australia. Questionnaires were administered to consenting patients utilising established HAE-specific and general survey instruments.

**Results:**

We identified 35 people with HAE in South Australia, yielding a population prevalence of 1 in 52,400, in line with average established international prevalence. HAE was identified in 4 patients of Indigenous Australian heritage. Seventeen of 31 adult patients completed an additional multi-questionnaire survey, revealing overall satisfactory disease control. Most common prophylactic therapies were danazol, lanadelumab, and subcutaneous C1 inhibitor. Many patients (mostly male) with milder disease had responded well to low-dose danazol with good tolerance and have continued to use it, whereas patients with higher disease burden are now using newer therapies, and overall satisfaction with current prophylaxis is high.

**Conclusions:**

Prevalence of HAE in South Australia aligns with international reports. Our population survey indicates that current long-term prophylaxis therapies including danazol, lanadelumab and C1-inhibitor, applied to appropriate patients taking into account disease activity and drug risks and tolerance, are effective for HAE attack prevention and produce high levels of satisfaction.

## Introduction

Hereditary angioedema due to deficiency (type 1) or defective function (type 2) of C1 inhibitor protein is caused by loss of function mutations in the SERPING1 gene which may be autosomal dominantly inherited or occur through de-novo gene mutation.^1^ Although the precise mechanism is unknown, haploinsufficiency of SERPING1 results in C1 inhibitor functional deficiency, with resultant dysregulated activation of factor XII and prekallikrein leading to excessive generation of bradykinin, which mediates vasodilatation and vasopermeability. Clinically HAE manifests as recurrent episodes of localised angioedema of cutaneous and mucosal regions, resulting in swelling episodes (“attacks”) which may be debilitating, painful and life-threatening if involving the airway.

HAE prevalence has been investigated in several studies with population frequency reported between 1 in 30,000 to 1 in 92,000,^2^, ^3^, ^4^, ^5^ varying by region and by study methodology. Estimates for de-novo mutational rate of 1 in 72,000,^2^ suggest that this may be a lower limit of disease prevalence. However, in Australia there have been no systematic studies assessing local prevalence of the condition. Furthermore, patient disease burden vs. disease control in the context of recent newly available therapeutic options has not been reported.

In this study we aimed to ascertain the prevalence of HAE in South Australia, a state with a population of 1.8 million, and undertake a survey of genealogy, HAE disease activity, quality of life, therapeutic experiences, and unmet needs in the cohort.

## Methods

This study was approved by the Central Adelaide Local Health Network Human Research and Ethics committee (Ref. 14221). The study was designed in 2 phases. Firstly, a multipronged ascertainment of all children and adults with HAE in South Australia, with recording of basic demographic parameters and current prophylactic therapy where possible from hospital and pharmacy records. Secondly, administration of a comprehensive multi-dimensional questionnaire to all consenting adults with review of clinical records for further information.

### Case ascertainment

**Fig. 1:**
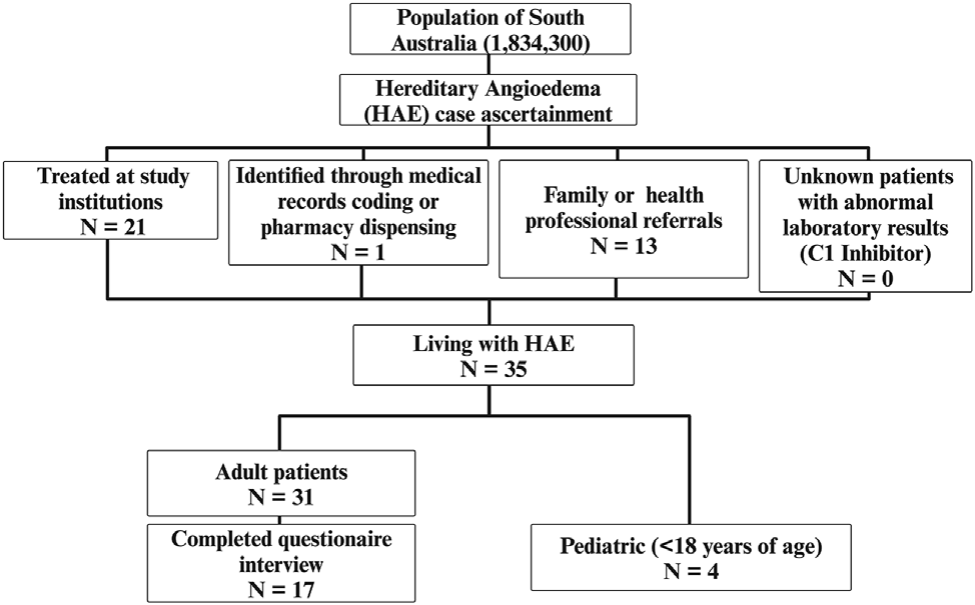
**Study design and case ascertainment.** South Australian population of hereditary angioedema (HAE) subjects were obtained through interrogation of medical records at the participating institution (diagnosis of HAE or presentation with angioedema), HAE specific therapy dispensing histories through public hospital pharmacies (Danazol, icatibant, C1-INH SC, lanadelumab), local health professionals both working in public and private sectors and in addition search of HAE specific diagnostic testing (C1 inhibitor quantitative and functional levels, the latter determined by chromogenic assays) through local public laboratory services

Several strategies were employed to ascertain cases (Fig. 1A). We recorded all known cases at our institution, Royal Adelaide Hospital Immunology Clinic. All immunologists in South Australia (based at Flinders Medical Centre and Women's and Children's Hospital) were notified through statewide meetings. Emails were sent to all dermatologists in South Australia through the state branch of the Australasian College of Dermatologists. Advertisements were placed in the General Practice Link section of the CALHN website. Patients were informed of the study through HAE Australasia. C1 Inhibitor functional and quantitative assay results at SA Pathology (statewide central laboratory) were reviewed for the past 10 years. Individuals who presented in the past 5 years to state hospitals with documented SNOMED and ICD-10 codes for hereditary angioedema, idiopathic angioedema, and associated codes with laboratory results for C1 inhibitor testing were screened for possible additional cases. When a previously unknown case was identified, medical records were reviewed, and where appropriate clinicians involved in care of patient were contacted for further information and referral for inclusion in study. Family members were considered and tested for HAE diagnosis where possible. Cases were counted if there was a consistent clinical history ascertainable and abnormal C1INH functional level (by chromogenic assay method). Cases with an isolated C4 level being low, in absence of further confirmatory testing were excluded.

Cases identified through screening were excluded when any of: repeated measures of C1 Inhibitor functional testing were normal in absence of any C1INH replacement therapy; case note records alternative diagnosis such as idiopathic angioedema, angiotensin converting enzyme inhibitor related angioedema, diagnosis of acquired angioedema or hereditary angioedema with normal C1 inhibitor (formally, Type 3); individual is deceased.

### Questionnaires

A multi-parameter interview questionnaire was designed, composed from 4 validated tools: hereditary angioedema quality of life questionnaire (HAE – QoL) which qualifies quality of life (QOL) over multiple domains within the past 6 months;^6^^,^^7^ HAE quality of life angioedema control test (AECT)^8^ which quantifies disease activity over the past 4 weeks; HAE-activity scale (HAE-AS).^9^ AECT and HAE-AS had validated cut-offs for low disease activity indicated to be a score above 9 and below 13 respectively, validated by each questionnaire.^8^^,^^9^ We also assessed occupational and educational impact using the Work Productivity and Activity Impairment (WPAI) instrument applied to hereditary angioedema, focussing on total disease related work impairment (referred to as ‘Total impairment’).^10^ In addition, specific historical questions were asked around diagnosis, treatments and satisfaction of treatments with the complete list of additional survey questionnaire presented in Supplementary File 1.

### Therapy questionnaires

Surveyed subjects were asked a structured questionnaire on each therapy they had been exposed to since their diagnosis, its subjective efficacy and their overall satisfaction with the therapy on a graduated visual analogue scale from 0 to 10 with zero being no satisfaction, dissatisfaction and 10 being complete satisfaction with therapy.

### Statistical analysis

Questionnaires were statistically analysed with Wilcoxon signed-rank test used for calculation of all p values with a statistical significance of 0.05 used by default. Bonferroni multiple comparisons was applied to inter-group analysis of questionnaires. All analysis and graph generation was conducted using R, version 4.3.1 and RStudio, version, 2023.03.0 + 386.

## Results

### Epidemiology of hereditary angioedema

Multimodal case ascertainment yielded 35 cases of confirmed hereditary angioedema from 8 kindreds as well as 7 individuals with no family history, representing a population prevalence of 1 in 52,400 peoples.^11^ Thirty one (89%) had type 1 HAE, 4 type 2. Four subjects from 3 independent family groups identified as Aboriginal or Torres strait islander (ATSI) heritage, representing a prevalence of HAE in ATSI populations of 1 in 14,200 in South Australia. Of the 35 subjects, 18 were male (51%) and the average age was 37 years (Table 1). At the cut-off date (end 2022) there were 4 children <18 years of age. Sixteen subjects were at the time of the survey on LTP with Danazol (3 of these were female and 2 postmenopausal), whilst 2 children were taking tranexamic acid and 4 people (2 of them children) were on no prophylactic therapy. The daily dose of danazol was low, at 100 mg in all subjects in whom data was reported (9 of 16). Demographic and basic therapeutic information provided in Table 1. Seventeen patients (50%) of the adult population (N = 31) agreed to participate in additional surveys. Mutations in the SERPING1 gene were found in 3 individuals from three families, c.31_317del (Pro105LeufsTer42), c.1439T > A (Val480Glu) and c.1396C > T (Arg466Cys) amongst surveyed patients (from 13 pedigrees). Three additional patients who did not complete the survey shared the c.31_317del mutation.

**Table 1:** Demographic and treatment summaries of HAE subjects in South Australia.

Characteristic	Overall, N = 35^a^	Unsurveyed, N = 18^a^	Surveyed, N = 17^a^
Current age	37 (4, 69)	27 (4, 69)	41 (20, 68)
Sex, Male	18 (51%)	10 (56%)	8 (47%)
HAE Type 1	31 (89%)	16 (89%)	15 (88%)
HAE Type 2	4 (11%)	2 (11%)	2 (12%)
**Current long-term prophylactic therapy**			
C1 INH SC	2 (5.7%)	0 (0%)	2 (12%)
Danazol	16 (46%)	7 (39%)	9 (53%)
Lanadelumab	10 (29%)	4 (22%)	6 (35%)
Tranexamic acid	2 (5.7%)	2 (11%)	0 (0%)
None	4 (11%)	4 (22%)	0 (0%)
Unknown	1 (2.9%)	1 (5.6%)	0 (0%)

a Median (Range); n (%). HAE, Hereditary Angioedema; C1 INH SC: C1 Inhibitor concentrate subcutaneous

### Diagnostic delays, ineffectual therapies, and attack locations

**Fig. 2:**
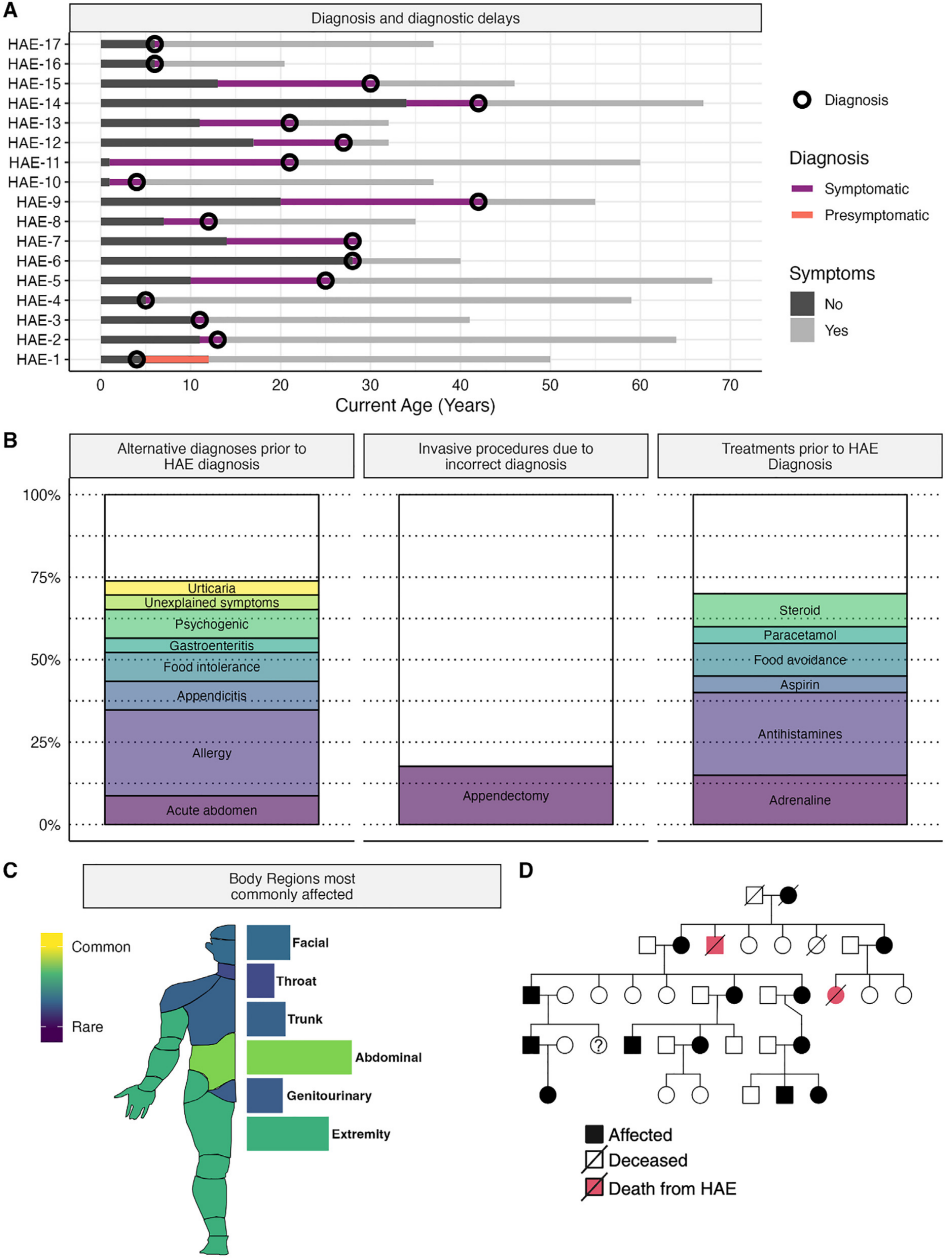
**Diagnostic delays, incorrect diagnoses and treatments and disease attack locations.** A) Surveyed subjects (N = 17) provided detailed histories of their diagnostic odyssey, with all but one being diagnosed pre-symptomatically. B) Majority of subjects were given an incorrect diagnosis and provided an ineffectual or incorrect therapy initially before correct diagnosis and 3 subjects underwent an Appendectomy. C) The most common location for attacks in our cohort were abdominal, and extremity with facial, genital and throat being much less common. D) HAE is a highly penetrant autosomal dominant disease with many kindreds having multiple generations affected and deaths from the disease in recent memory

All but 1 of 17 surveyed individuals experienced a diagnostic delay (Fig. 2A), with a median diagnostic delay of 11.5 years (range 2–22 years) from symptom onset for those who were not diagnosed pre-symptomatically. Incorrect diagnoses were made initially in 11 (65%) of patients with 3 (17%) undergoing a needless appendectomy (Fig. 2B). Abdominal attacks, followed by extremities, face, trunk, and genitourinary were most common locations to experience angioedema symptoms (Fig. 2C), with airway or throat attacks being the least commonly reported. In 8 of the surveyed kindreds, a family member had been known to have died from a fatal angioedema attack.

### Angioedema activity, quality of life, and productivity questionnaires

**Fig. 3:**
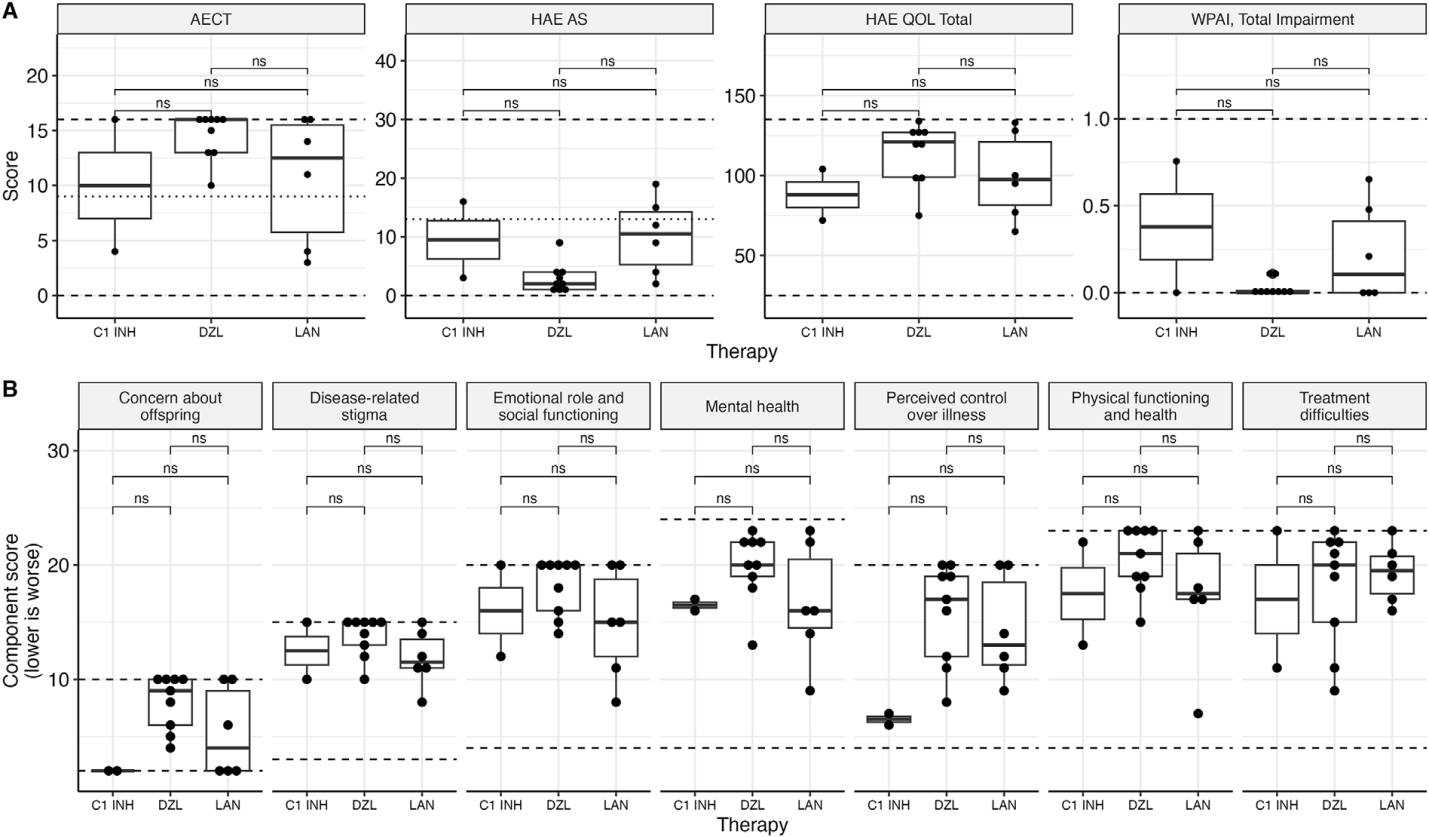
**Quality of life, disease activity and productivity scores in N** = **17 surveyed HAE subjects by current HAE prophylaxis therapy.** A) Angioedema control test (AECT), Hereditary Angioedema Activity Score (HAE-AS), HAE – Quality of life (HAE-QOL) questionnaire and Work productivity activity index, total workplace productivity impairment (WPAI). B) 7 subdomains of HAE-QOL. Dashed lines represent the upper and lower bounds for each questionnaire and dotted line (AECT and HAE-AS only) indicates the cut-off for low disease activity as judged by each tool. Pairwise Wilcoxon tests were applied to assess for statistical difference, applying Bonferroni correction for multiple comparisons. C1INH, C1-Inhibitor Concentrate; DZL, Danazol; LAN, Lanadelumab

Four validated, structured questionnaires were applied to the surveyed cohort and subjects were grouped based on their current prophylactic therapy to investigate any impact of traditionally considered lower-efficacy therapies on QOL and disease activity. Strikingly, subjects on danazol indicated good overall QOL, as well as low HAE disease activity as measured by AECT and HAE-AS (Fig. 3). One female subject on C1 Inhibitor concentrate, subcutaneous (C1-INH SC) and 2 females on lanadelumab indicated poor disease control on both questionnaires (Fig. 3), though all had only recently commenced on these therapies having previously used other agents (tranexamic acid in the case of C1INH-SC patient, danazol, and C1INH-SC for those now on lanadelumab).

### Therapeutic odyssey and treatment satisfaction

**Fig. 4:**
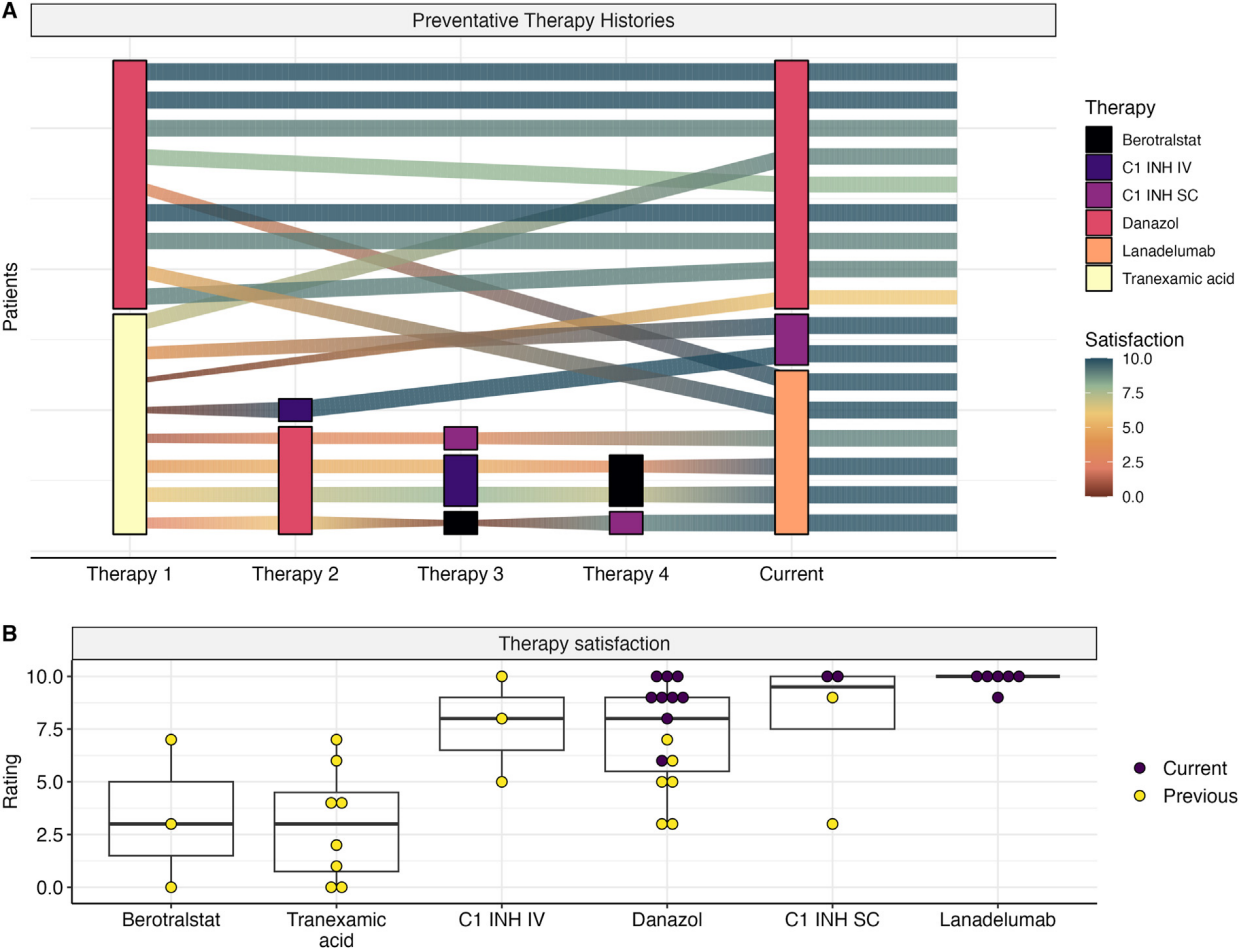
**Therapeutic odyssey and treatment satisfaction**. A) Therapeutic histories of subjects N = 17 displayed as a network graph, Sankey-plot showing progress through therapies. Connecting line colour and width demonstrate satisfaction rating of the previous nodes therapy. B) Boxplot of therapy satisfaction ratings with previous and current therapies, demonstrating progressive increase in therapeutic satisfaction with current versus previous therapies (For interpretation of the references to colour in this figure legend, the reader is referred to the Web version of this article.)

Drug histories and therapy satisfaction ratings were obtained from all surveyed subjects (Fig. 4A). Amongst responding participants, there was a mean of 1.3 prior therapies (median of 1), with 7 subjects only ever having taken danazol for prophylaxis. For patients currently on lanadelumab or C1-INH SC, an average of 2.2 prior therapies were trialled. Satisfaction broadly improved after all therapies, with a notable exception of berotralstat, accessed through a clinical trial, which was associated with poor satisfaction ratings, on par with tranexamic acid. Of those patients on lanadelumab, 4 had previously used C1-INH either subcutaneously or intravenously and indicated an improvement in treatment satisfaction after change to their current treatment.

## Discussion

In this paper we report the first study of HAE prevalence in Australia. We attempted to identify all individuals with HAE through a variety of ascertainment methods and whilst there may be other cases missed, our figure represents a minimum and probably close to accurate prevalence figure, and is in line with international reports. In our cohort 4 individuals identified as aboriginal or Torrens strait islander (ATSI), providing a population prevalence which is three times higher than in the non-indigenous. This represents the first report of HAE prevalence in ATSI peoples, where founder effects^12^ and poor access to genetic services^13^ may result in higher incidence and diagnostic delay. Therefore, in patients of indigenous heritage presenting with angioedema, HAE should be considered.

Historically, HAE sufferers incurred a high burden of disease, with high levels of depression, anxiety and absenteeism.^14^, ^15^, ^16^, ^17^ The unpredictable nature of attack occurrence as well as frequency and severity of attacks have been identified as a key factors causing impaired quality of life.^18^ Past therapies for HAE included the androgen danazol, the plasminogen antagonist tranexamic acid (TXA), and fresh frozen plasma (FFP). Danazol can be highly efficacious for some patients but may not be tolerated due to side effects and carries long-term risks;^19^ hence, it has fallen out of favour and is not used at all in many countries. In our cohort, low-dose danazol was associated with high participant satisfaction in some males and post-menopausal females, with limited perceived androgenic adverse effects, and as an oral route medicine, is consistent with known therapeutic preferences in HAE patients.^20^ This is, of course, a result of selection since those who required a higher dose to achieve control, or who did not tolerate the drug, would have been transferred to a different therapy. Thus, our study reiterates that danazol may be an option in certain patients, and that there remains a need for simple, accessible, oral prophylaxis options, with good risk profiles.

Over the past 2 decades numerous effective and targeted therapies for HAE have emerged including regular prophylactic intravenous or subcutaneous donor derived or recombinant C1 INH, inhibitors of kallikrein (berotralstat, lanadelumab, and ecallantide) and the bradykinin receptor antagonist icatibant. Appropriate usage of highly effective therapies, where available, may now facilitate near normal quality of life with potential for prevention of most or all attacks and access to effective acute therapies in case of breakthrough. In Australia, access to government-funded expensive prophylactic agents such as C1 INH and lanadelumab has been restricted to patients meeting threshold attack frequency criteria.

Despite availability of newer therapies, danazol remained the most frequently prescribed HAE prophylaxis in our cohort, with recipients having high therapeutic satisfaction and low disease activity. Notably, however, danazol access in Australia was restricted by discontinuation of supply immediately prior to commencement of this study,^21^ which likely contributed to the relatively disproportionate increase in treatment difficulties as judged by the HAE-QOL domain (Fig. 3E), although availability was re-established by special access programs. Australian guidelines mandate regular monitoring of patients on danazol for adverse effects.^22^ On the contrary, other oral therapies like tranexamic acid and berotralstat (available through a clinical trial with open label extension) in our cohort were generally considered ineffective and unsatisfactory, in patients with high disease activity (as required for involvement in the trial). Lanadelumab, which became available for poorly controlled HAE in Australia immediately prior to the study period (December 2021), showed a rapid adoption, largely in subjects on C1 Inhibitor concentrates (C1-INH) or with poor disease control, aligning with expectations. For women, danazol's frequent virilising effects compound on other adverse effects such as hepatic adenomas, rarely carcinomas and liver function abnormalities, headaches and psychological disturbances.^19^ Additionally, estrogens are known to contribute to disease activity by increasing activity of bradykinin and fibrinolytic pathways,^23^ summating to danazol being less well tolerated and less effective especially at low dose. Consequently, a majority of lanadelumab users in our study (6 of 10) were premenopausal females.

**Supplemental Fig. 1:**
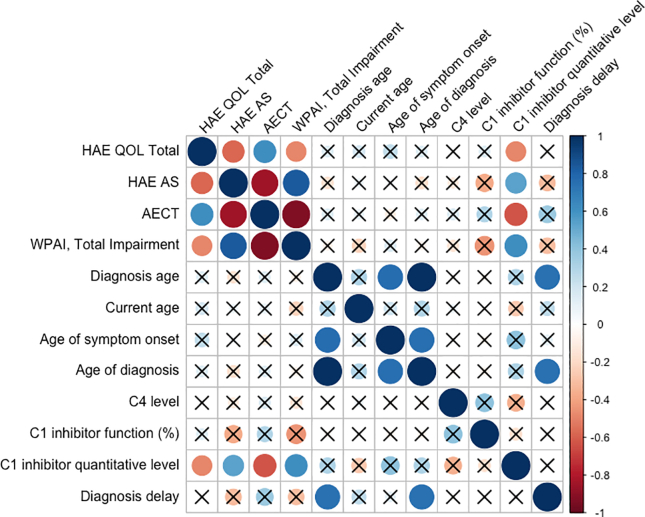
Correlation of study variables including demographics, laboratory indices, QOL questions and angioedema activity scores. Strong inter-questionnaire correlation observed between HAE-QOL, HAE-AS, AECT and WPAI measures. Colour and circle size indicates direction and magnitude of agreement, crossed out circles are statistically non-significant

Quality of life measures across our cohort are comparable with initial validation and subsequent studies in HAE. Overall median scores for WPAI were on par with end of study measures in the COMPACT extension study (WPAI total impairment, 14% (SD 25%) in this study (Table 2) vs 9.45%, SD 21%).^24^ Similarly, HAE-QOL is on par with the end of treatment Self-Administered C1-INH SC In Hereditary Angioedema (SABHA) study,^25^ though in this study the HAE-QOL did not differ significantly before and after treatment. Critical analysis of the study design and the questionnaires applied identified very clear and close inter-questionnaire correlation between all administered disease activity, productivity and QOL scores (Supplemental Fig. 1). This suggests that future studies may consider a single, comprehensive questionnaire to assess disease activity in one snapshot, though acknowledging that QOL impairment does not necessarily correlate with disease control scores as highlighted by Bork et al.^26^ However, a limitation of our study is the (fortunate) under-representation of patients with poor disease control, which clearly skews our measures, though reflects the realisation of effective therapies now available for HAE in Australia. Overall, we conclude that disease activity in our HAE cohort is low, on par with international interventional studies, and minimally impacts productivity.

**Table 2:** Quality of life and disease activity questionnaire results by prophylaxis.

Characteristic	Overall, N = 17^a^	Berinert SC, N = 2^a^	Danazol, N = 9^a^	Lanadelumab, N = 6^a^
HAE QOL Total	106 (65, 134)	88 (72, 104)	114 (75, 134)	100 (65, 133)
HAE AS	6.3 (1.0, 19.0)	9.5 (3.0, 16.0)	3.0 (1.0, 9.0)	10.2 (2.0, 19.0)
AECT	12.6 (3.0, 16.0)	10.0 (4.0, 16.0)	14.6 (10.0, 16.0)	10.7 (3.0, 16.0)
WPAI, Total Impairment	0.14 (0.00, 0.76)	0.38 (0.00, 0.76)	0.03 (0.00, 0.12)	0.22 (0.00, 0.65)

a Mean (Range)

Nonetheless, subjects with HAE desire further improvements in therapies, as highlighted by the concern and feelings of regret participants reported in passing on the disease to future generations. Thus, future therapies including ultra-long acting RNA-based inhibitors of prekallikrein^27^ and C1INH gene therapy^28^ may further improve the quality of life of patients with HAE.

Our study has several strengths-firstly comprehensive case ascertainment likely identified most if not all HAE cases in our population. Secondly, through comprehensive interviews of a high proportion of adults, we could obtain a very clear snapshot of the current impact of HAE and its management on our population. Limitations of this study include incomplete recruitment of subjects to the questionnaire potentially biasing the data to subjects more willing to provide feedback. Secondly, it should be noted our cohort is from a resource-rich setting with specific therapeutic availabilities and histories.

In conclusion, the prevalence of HAE in South Australia is similar to international quoted figures, with a notable occurrence in Indigenous Australians. HAE patients have a range of therapeutic options in Australia and patients with frequent and severe attacks can access recent effective prophylactic agents, while danazol remains an effective and popular orally-administered therapy for those with milder disease who require only low dose, with good tolerance, for disease control. Future therapies should prioritize continued reduction in the burden of treatment and further normalise quality of life in this population.

## Abbreviations

ATSI, Aboriginal and Torrens Strait islander; C1 INH, C1 Inhibitor concentrate; HAE, Hereditary Angioedema; QOL, Quality of Life; SC, Subcutaneous

## Acknowledgements

Authors wish to thank patients and their family members.

## Funding

This study was funded by Allergy and Immunololgy Foundation of Australasia (10.13039/501100003197AIFA) Primary Immunodeficiency Grant.

## Availability of data and materials

De-identified, original data is available on request to corresponding author.

## Author contributions

AT, KM, PH. AL and WS contributed to study design and production of the manuscript. AT and KM and AL performed data collection. AT performed Data analysis and generated figures and graphs.

## Ethics approval

This study was approved by the Central Adelaide Local Health Network Human Research and Ethics committee (Ref. 14221).

## Authors' consent to publication

All authors consent to this publication.

## Declaration of competing interest

WS has received consulting fees from 10.13039/100008322CSL Behring and non-financial support from Takeda. All other authors declare no relevant conflicts of interest.

## Appendix A Supplementary data

Supplementary File 1 Additional Questions asked in the semi-structured interview. Published Quality of Life and Productivity tools are not included but available from their respective sources. Supplementary File 1
